# Polyploid QTL‐seq towards rapid development of tightly linked DNA markers for potato and sweetpotato breeding through whole‐genome resequencing

**DOI:** 10.1111/pbi.13633

**Published:** 2021-06-02

**Authors:** Hiromoto Yamakawa, Emdadul Haque, Masaru Tanaka, Hiroki Takagi, Kenji Asano, Etsuo Shimosaka, Kotaro Akai, Satoshi Okamoto, Kenji Katayama, Seiji Tamiya

**Affiliations:** ^1^ Institute of Crop Science National Agriculture and Food Research Organization (NARO) Tsukuba Ibaraki Japan; ^2^ Kyushu‐Okinawa Agricultural Research Center National Agriculture and Food Research Organization (NARO) Miyakonojo Miyazaki Japan; ^3^ Department of Bioproduction Science Ishikawa Prefectural University Nonoichi, Ishikawa Japan; ^4^ Hokkaido Agricultural Research Center National Agriculture and Food Research Organization (NARO) Memuro Hokkaido Japan; ^5^ Present address: Center for Seeds and Seedlings National Agriculture and Food Research Organization (NARO) Tsukuba Ibaraki Japan; ^6^ Present address: Tohoku Agricultural Research Center National Agriculture and Food Research Organization (NARO) Morioka Iwate Japan

**Keywords:** NGS mapping, QTL‐seq, potato, sweetpotato, polyploid, DNA marker

## Abstract

Potato (*Solanum tuberosum* L.) and sweetpotato (*Ipomoea batatas* L.), which are nutritionally and commercially important tuberous crops, possess a perplexing heredity because of their autopolyploid genomes. To reduce cross‐breeding efforts for selecting superior cultivars from progenies with innumerable combinations of traits, DNA markers tightly linked to agronomical traits are required. To develop DNA markers, we developed a method for quantitative trait loci (QTL) mapping using whole‐genome next‐generation sequencing (NGS) in autopolyploid crops. To apply the NGS‐based bulked segregant method, QTL‐seq was modified. (1) Single parent‐specific simplex (unique for one homologous chromosome) single‐nucleotide polymorphisms (SNPs), which present a simple segregation ratio in the progenies, were exploited by filtering SNPs by SNP index (allele frequency). (2) Clusters of SNPs, which were inherited unevenly between bulked progenies with opposite phenotypes, especially those with an SNP index of 0 for the bulk that did not display the phenotypes of interest, were explored. These modifications allowed for separate tracking of alleles located on each of the multiple homologous chromosomes. By applying this method, clusters of SNPs linked to the potato cyst nematode resistance *H1* gene and storage root anthocyanin (AN) content were identified in tetraploid potato and hexaploid sweetpotato, respectively, and completely linked DNA markers were developed at the site of the presented SNPs. Thus, polyploid QTL‐seq is a versatile method that is free from specialized manipulation for sequencing and construction of elaborate linkage maps and facilitates rapid development of tightly linked DNA markers in autopolyploid crops, such as potato and sweetpotato.

## Introduction

Potato (*Solanum tuberosum* L.) and sweetpotato (*Ipomoea batatas* L.) are important crops not only for serving as staple foods in developing countries but also in a variety of cuisines worldwide. However, the clonal propagation of these crops occasionally spreads their pathogens and pests rapidly, leading to devastating damage to their production areas. Therefore, breeders are confronting pressure to continuously develop new cultivars with enhanced resistance to them (Katayama et al., 2017; Mori et al., 2015). On the other hand, potato and sweetpotato are superior in nutrition because they are rich in vitamins, minerals and fibre. In particular, purplish sweetpotato is gaining attention in terms of the health‐promoting function of anthocyanins (AN) (Tanaka et al., 2017). Additional efforts are being made to breed varieties of coloured sweetpotato (Tanaka et al., 2019).

Considering the breeding of these tuberous crops, their heterologous autopolyploidy, in which each homologous chromosome has different origin by outcross with different sequences, poses an inevitable issue of complicated heredity. In the case of a diploid crop, rice, the combination of 12 chromosomes in the F_2_ progeny generated by a cross of two different parents is 5.3 × 10^5^, which was calculated by 3^12^ [3 distribution patterns (AA, AB, BB) for each of 12 chromosomes], without considering crossovers among homologous chromosomes. Assuming that each individual has a different combination of chromosomes, a few hectares of paddy fields are sufficient for planting all combinations. In contrast, the number of combinations of tetraploid potato and hexaploid sweetpotato F_1_ progenies after crossing is 4.7 × 10^18^ and 1.1 × 10^39^, which were calculated by (_4_ *C*_2_ × _4_ *C*_2_)^12^ and (_6_ *C*_3_ × _6_ *C*_3_)^15^, respectively, where *_n_C_r_* means combination in case that *r* chromosomes were chosen from *n* different homologous chromosomes of one parent and transmitted to progenies. Those would require thousands of the earth’s surfaces to cultivate all the combinations. Therefore, DNA marker‐assisted selection is imperative in selecting superior individuals from such innumerable combinations efficiently before planting to fields.

DNA markers of agronomically important traits, such as disease and pest resistance, have been previously developed in potato (Asano and Tamiya, 2016; Dalamu et al., 2012). Among them, sets of markers linked to the potato cyst nematode resistance gene, *H1*, are widely incorporated in current breeding programmes (Asano et al., 2012). However, in sweetpotato, the availability of DNA markers for practical breeding is limited. Recently, the reference genome sequences of homozygous, doubled‐monoploid potato (Hirsch et al., 2014; Pham et al., 2020; Potato Genome Sequencing Consortium, 2011; Sharma et al., 2013) and diploid relatives of sweetpotato (Hirakawa et al., 2015; Li et al., 2019; Wu et al., 2018) were released. They pave the way for rapid mapping of quantitative trait loci (QTLs) and the development of DNA markers on such polyploid crops through next‐generation sequencing (NGS)‐based high‐throughput genotyping methods that utilize numerous genome‐wide single‐nucleotide polymorphisms (SNPs). Resistance gene enrichment sequencing (RenSeq) and generic‐mapping enrichment Sequencing (GenSeq) are mapping‐by‐sequencing methods using sequence‐captured libraries, which target arbitrarily selected nucleotide binding‐site leucine‐rich repeat resistance genes, and single/low‐copy number genes dispersed throughout the potato genome, respectively. Using these methods, DNA markers linked to late blight resistance in diploid potato (Chen et al., 2018), and potato cyst nematode resistance in tetraploid potato (Strachan et al., 2019) have been developed. In sweetpotato, restriction site‐associated DNA sequence (RAD‐seq)‐based mapping methods have been developed to identify QTLs for root‐knot nematode resistance (Oloka et al., 2021; Sasai et al., 2019), AN and β‐carotene content in coloured storage roots (Gemenet et al., 2020; Haque et al., 2020a; Haque et al., 2020b), and yield (Okada et al., 2019). Both sequence capturing and RAD‐seq‐based methods reduced the time and effort required for the development of DNA markers. However, complicated procedures to prepare libraries appropriate for sequencing and subsequent construction of genetic linkage maps prevent breeders from incorporating these advanced techniques for breeding materials newly generated by crossing different sets of parents. Furthermore, since arbitrarily selected SNPs are exploited for mapping in these methods, there might be cases where SNPs located in the vicinity of agronomically important genes escape from the analyses and tightly linked DNA markers are not developed. Therefore, a method that is handy (free from specialized manipulation for sequencing and the construction of elaborate linkage maps) but is robust (providing tightly linked markers), is required.

In autopolyploid species, simplex polymorphism, where only one chromosome has a nucleotide base different from that of the reference sequence among multiple homologous chromosomes, is exclusively used for genetic mapping, since it segregates in the progeny with simple ratios of simplex:nulliplex = 1:1 (in case one parent has simplex) or duplex:simplex:nulliplex = 1:2:1 (in cases where both parents have the same nucleotide simplex). Therefore, accurate discrimination of simplex polymorphism is a prerequisite for mapping QTLs in crops with polyploid genomes. To accurately distinguish simplex from duplex, many sequence reads are necessary for each SNP site. RenSeq/GenSeq and RAD‐seq, which provide extensive sequencing of selected regions of the genome, generate a large number of reads for the selected SNP sites, allowing discrimination of simplex with sufficient accuracy. However, they fail to detect a large proportion of SNPs, which omits them for sequencing. In contrast, the QTL‐seq method developed by Takagi et al. (2013) is intended to identify QTLs by whole‐genome sequencing. In this method, all SNPs dispersed throughout the genome are exploited. However, sequencing the whole genome with the same amount of read data without arbitrary selection reduces the number of reads per SNP site, leading to ambiguous discrimination of the simplex.

Recently, the cost of whole‐genome sequencing has been tremendously reduced, and the performance of computers continuously increases. Therefore, analyses with massive data of whole‐genome sequences are feasible as was the case of an allopolyploid crop, peanut (Clevenger et al., 2018). In this study, we aimed to develop a method for QTL analysis by whole‐genome resequencing with sufficient amounts of read data in autopolyploid crops by dealing with progenies segregating the presence of the *H1* gene in potato and tuber AN content in sweetpotato as model cases. We propose the polyploid QTL‐seq method as a versatile tool for rapid development of tightly linked DNA markers.

## Results

### Development of polyploid QTL‐seq

For the purpose of genetic mapping of QTLs in autopolyploid species, simplex polymorphism is generally used because of the simplicity of segregation patterns in the progeny after crossing. A certain number of reads are necessary for the accurate extraction of simplex SNPs. Therefore, a simulation test was conducted to determine the number of reads (depth) required. In tetraploid species such as potato, the frequencies of simplex, duplex and triplex SNPs were selected and read by random shotgun sequencing, namely SNP indices, converged to 0.25, 0.50 and 0.75, respectively, as read depth increased. Simulation of 10,000 replications revealed that a depth of 40 is sufficient to distinguish simplex from duplex with 95% confidence by extracting SNPs with SNP index ranges of 0.10 to 0.36 (Figure S1). In hexaploid species such as sweetpotato, SNP indices of simplex, duplex, triplex, tetraplex and pentaplex approached 0.17, 0.33, 0.50, 0.67 and 0.83, respectively, as read depth increased. Simulation of 10 000 replications suggested that a depth of 75 is necessary to distinguish simplex from duplex with 95% confidence by extracting SNPs with SNP index ranges of 0.08 to 0.25 (Figure S1).

**Figure 1:**
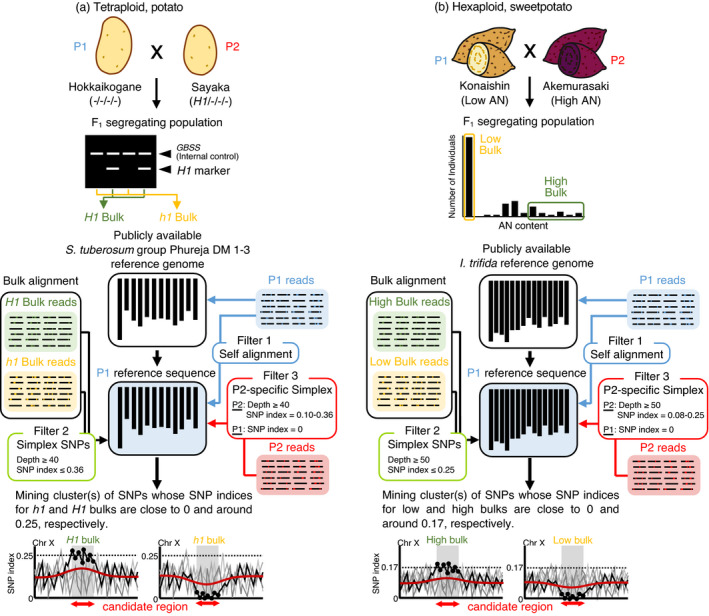
Schematics of the polyploid QTL‐seq. (a) Identification of the potato *H1* locus. Tetraploid potato cultivars, Sayaka (P2, *H1* simplex) and Hokkaikogane (P1, nulliplex) were crossed to generate a F_1_ population segregating *H1*. DNA samples extracted from *H1*‐haboring and *H1*‐absent individuals were separately pooled to prepare two bulked DNA samples, designated *H1* and *h1* bulks respectively, and subjected to NGS. The *H1* locus were identified by the following filtering strategy. NGS reads obtained for one of the parents (P1 in this case) were aligned to the publicly available reference genome sequence to prepare the P1 reference sequence by replacing nucleotides with the majority bases of the SNP index (the ratio of reads containing a certain SNP to the total number of reads covering a given SNP position) of >0.5 at all positions. Then, *H1* and *h1* bulk reads were independently aligned to the P1 reference sequence to calculate SNP index values. The following filters were applied to select P2‐specific simplex SNPs: (1) self‐alignment (P1 reads aligned to P1 reference sequence) to remove spurious SNPs, (2) extraction of confidential simplex SNPs with depth of ≥40 and SNP index of ≤0.36, which is the expected value for a combination of nulliplex and simplex individuals on alignment of both bulk reads, and (3) extraction of the P2‐specific simplex by choosing SNPs with depth of ≥40 and SNP index of 0.10–0.36 (an expected range for simplex) on P2 read alignment. For identification of candidate loci, cluster(s) of SNPs, whose SNP indexes for *h1* and *H1* bulks are close to 0 and around 0.25, respectively, were explored. (b) Detection of sweetpotato purplish AN content QTL(s). Hexaploid sweetpotato cultivars, Konaishin (P1, low AN) and Akemurasaki (P2, high AN), were crossed to generate an F_1_ population segregating AN content. DNA samples were extracted and pooled to prepare low and high bulks according to AN content of F_1_ individuals. QTL‐seq and the filtering strategy were the same as the potato *H1* case (a) except for exploitation of depth of ≥50 and SNP index of ≤0.25 to extract simplex SNPs.

Following the above simulation, the QTL‐seq algorithm was modified with three filtering strategies to adapt to the polyploid genome (Figure 1). QTL‐seq combines bulked segregant analysis and whole‐genome resequencing for rapid identification of the genomic regions that differ between the two crossed parents and contribute to the trait of interest among the resulting progeny (Itoh et al., 2019; Takagi et al., 2013). In the analysis, frequencies in which SNPs were detected among reads aligned at given sites (i.e. SNP indices), were calculated and subjected to an analysis of correlation to the phenotype of interest. The algorithm started with the alignment of reads of either of the parents (herein designated P1), to a publicly available reference genome to prepare P1 reference sequences by replacing the detected SNPs. Then, the P1 reads were aligned again to the yielded P1 reference sequence to remove the detected SNPs from the following analysis as spurious SNPs (filter 1). Subsequently, the reads of bulked samples showing opposite phenotypes were aligned to the P1 reference sequence to calculate the SNP indices of both bulks. Then, simplex SNPs were extracted by an additional filter that permits SNPs with a depth of ≥40 and SNP index of ≤0.36, in case of tetraploid potato, or depth of ≥50 and SNP index of ≤0.25 in case of hexaploid sweetpotato for both bulks (filter 2), since reads corresponding to approximately 50‐time depth mapping were acquired in the present study. In sweetpotato, the use of SNPs with ≥50‐depth, which is a less strict threshold than the above 95% confidence estimation, which requires ≥75‐depth, might increase the possibility of unintended incorporation of duplex SNPs into the analysis, but it prevented a large proportion of simplex SNPs from being excluded from the analysis, and allowed QTL identification with as many SNPs as possible. Finally, reads of the other parent, designated P2, were aligned with the P1 reference sequence to calculate SNP indices. Then, P2‐specific simplex SNPs were selected by another filter that retained SNPs with a depth of ≥40 and an SNP index of 0.10–0.36 in case of potato, or depth of ≥50 and SNP index of 0.08–0.25 in case of sweetpotato for the P2 read, as well as an SNP index of 0 for the P1 read (filter 3).

Assuming that the trait of interest is regulated by an allele located on a single homologous chromosome, SNPs presenting linkage to the phenotype are limited to those located on the chromosome where the causal allele resides (black lines in the lower graphs of Figure 1, presenting inclined distribution of SNP index between two bulks around the candidate region), whereas the SNPs located on the other chromosomes do not show any linkage, even though they are in the vicinity of the QTL (grey lines, presenting no inclination). Such unlinked SNPs hinder the detection of QTLs by dampening skewed peaks of the SNP index when the average of their SNP indices within a certain region is calculated by the sliding window analysis (red lines). To overcome this dilution problem in the mapping of polyploid species, the SNPs with skewed SNP indices, particularly those close to 0 (0 to 0.02), for the bulk showing the same phenotype as P1 (potato *h1* and sweetpotato low AN bulks in this study), were counted on the list of P2‐specific simplex SNPs, and their distribution throughout the genome is shown as a histogram.

### Polyploid QTL‐seq of potato *H1*‐segregating progeny

**Figure 2:**
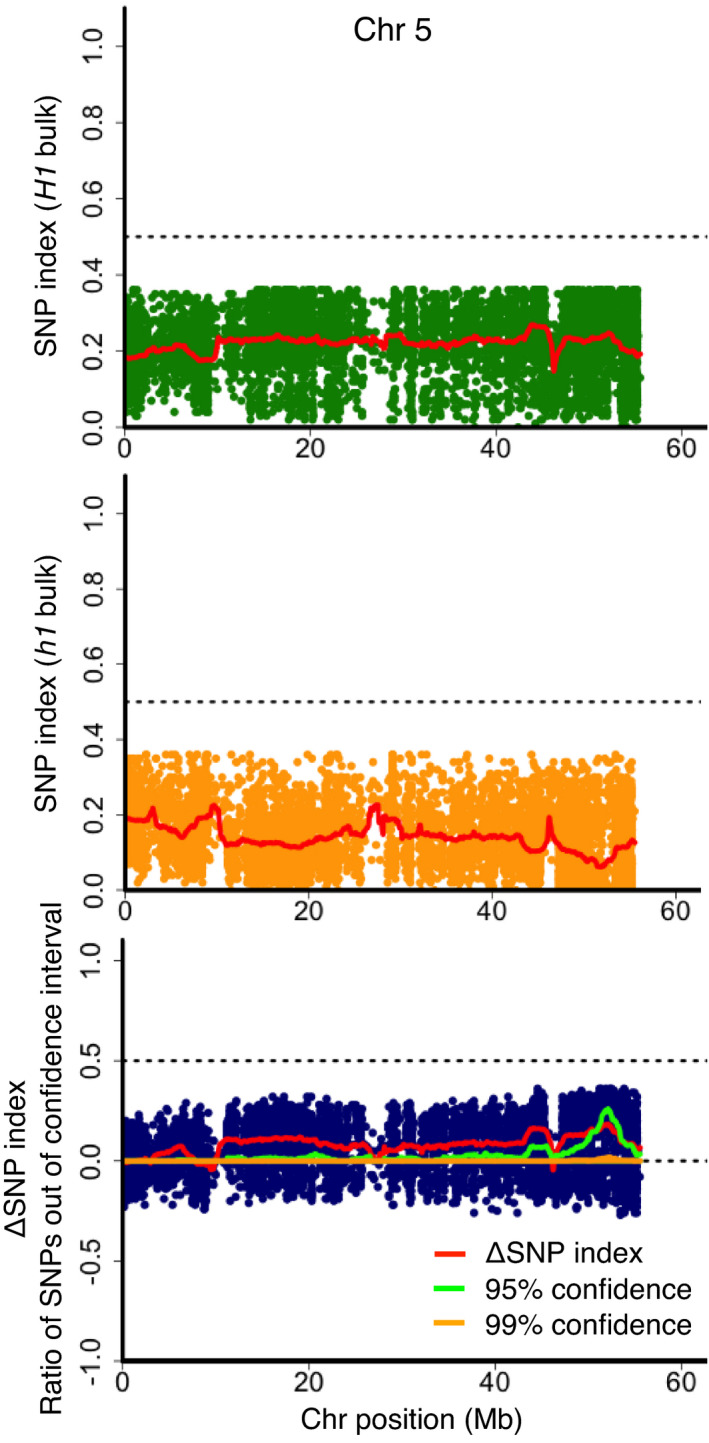
Identification of a genomic region corresponding to potato *H1* locus. SNP index plots of *H1* bulk (top) and *h1* bulk (middle), and ΔSNP index plot with ratio of SNPs out of 95% (green line) and 99% (orange line) statistical confidence intervals under the null hypothesis of no QTLs (bottom) are depicted for Chromosome 5. Red lines indicate the sliding window average of a 2 Mb interval with a 50 kb increment for SNP index and ΔSNP index.

**Figure 3:**
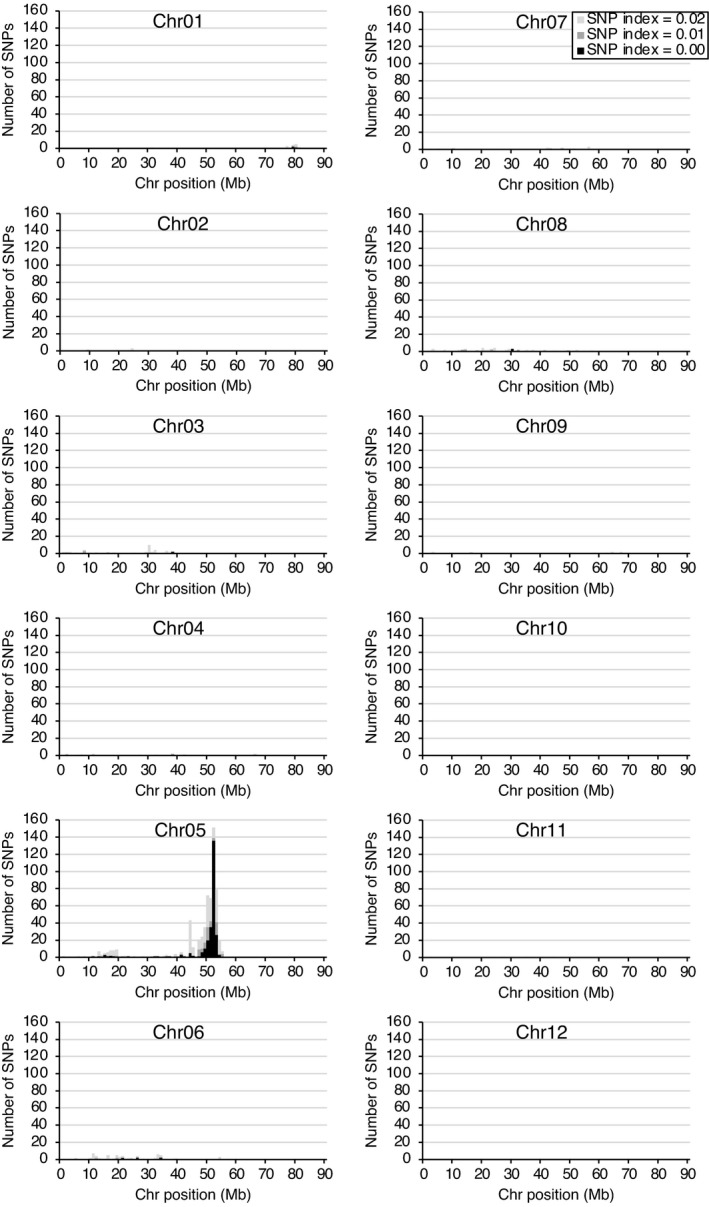
Identification of a genomic region corresponding to the potato *H1* locus by cluster analysis. For identification of the candidate loci, SNPs, whose SNP index for the *h1* bulk was 0, 0.01 and 0.02, were extracted from the Sayaka‐specific simplex SNPs (Table S1, *n* = 100 240), and their distributions throughout the whole chromosomes are indicated by black, grey and light grey, respectively.

As proof of principle, the above polyploid QTL‐seq algorithm was validated with a tetraploid potato F_1_ population segregating potato cyst nematode resistance gene, *H1*. Generally, polyploid QTL‐seq requires two reciprocal sets of analyses with simplex SNPs specific to each parent. However, in this analysis, cv. Sayaka is known as a donor of *H1*; thus, Sayaka reads were mapped against the reference augmented with SNPs from cv. Hokkaikogane. Mapping with 110.5 Gb, 114.4 Gb, 136.2 Gb and 107.7 Gb reads of Sayaka, Hokkaikogane, *H1* bulk and *h1* bulk (each consisting of 23 F_1_ individuals), corresponding to 49.2, 50.6, 47.9 and 48.8 times the coverage of the potato genome, respectively, yielded 100 240 simplex SNPs that were specific for the *H1*‐harbouring cultivar, Sayaka. Polyploid QTL‐seq with the simplex SNPs detected an increased number of SNPs that were skewed over the 95% statistical confidence in the region of chromosome 5, 47–54 Mb (as shown by the green line of the ΔSNP index graph in Figure 2). Even though the average SNP index, indicated by a red line, did not reach 0 in the *h1* bulk in this region (Figure 2), an extensive cluster of SNPs (whose SNP indices were 0 for the *h1* bulk) was manifest around 51.9–52.8 Mb (Table S1). Meanwhile, alignment of the *H1* bulk reads gave no such clusters of skewed SNPs. Although another cluster of SNPs with an SNP index close to 0 in the *h1* bulk was found in 12–20 Mb of the same chromosome, it did not consist of many SNPs with over 95% confidence. Since no other cluster showing such *h1* bulk‐specific distortion of the SNP index with confidence was detected throughout the whole genome (Figure S2), chromosome 5, 47–54 Mb was suggested to be the sole candidate region for the *H1* locus. Counting the number of SNPs whose SNP indices of the *h1* bulk were approaching 0 within each 1 Mb window also demonstrated that most of the SNPs with SNP indices below 0.02, were located in the region of chromosome 5, 47–54 Mb (Figure 3). In contrast, when polyploid QTL‐seq was conducted by exploiting the other parent, Hokkaikogane‐specific simplex SNPs with the reference genome generated by Sayaka reads, a small number of SNPs were detected as those skewed over 95% confidence (19) and those with SNP indices of 0 in the *h1* bulk read (14), and they were located sparsely throughout the genome (Figure S3), confirming that *H1* is transmitted to F_1_ progenies by a Sayaka‐derived homologous chromosome. Given the larger number of SNPs detected with Sayaka‐specific simplex SNPs, 378 and 300 for those skewed over 95% confidence and those with SNP index of 0 in the *h1* bulk, respectively, compared to those detected with Hokkaikogane‐specific simplex SNPs, 19 and 14 (*P* < 0.001 in chi‐square test following correction by total number of the detected SNPs, 100 240 and 71 083 for Sayaka‐specific and Hokkaikogane‐specific simplex, respectively), the polyploid QTL‐seq procedure was used to detect a trait‐controlling locus efficiently in the tetraploid crop, potato.

To reduce the sequencing cost for polyploid QTL‐seq, the effect of reduction in input read numbers on the efficiency of QTL detection was examined. As the numbers of parent and bulk reads were reduced to a half (52.5 Gb) and a quarter (26.25 Gb) of the original analysis, the thresholds of the read depth to select simplex SNPs were decreased from the original parameter, ≥40, to ≥25 and ≥15 to retain a large proportion of SNPs available for the analysis, respectively. Even though the reads of *H1* and *h1* bulks as well as those of both parents were reduced by half (52.5 Gb), the *H1* locus could be identified ˜47–54 Mb of chromosome 5 (Figure S4), although a certain number of SNPs with an SNP index of 0 for the *h1* bulk were detected on the other chromosomes, such as chromosomes 1, 6 and 8. However, further reduction in the input reads to one quarter (26.25 Gb) decreased the number of available Sayaka‐specific simplex SNPs to 33 609, approximately one third of the number in the original analysis with full reads, and the candidate peak on chromosome 5 was dampened to a level indistinguishable from other background peaks. Therefore, at least 50 Gb reads are necessary to detect the *H1* locus in potato.

### Polyploid QTL‐seq of sweetpotato storage root AN content‐segregating progeny

**Figure 4:**
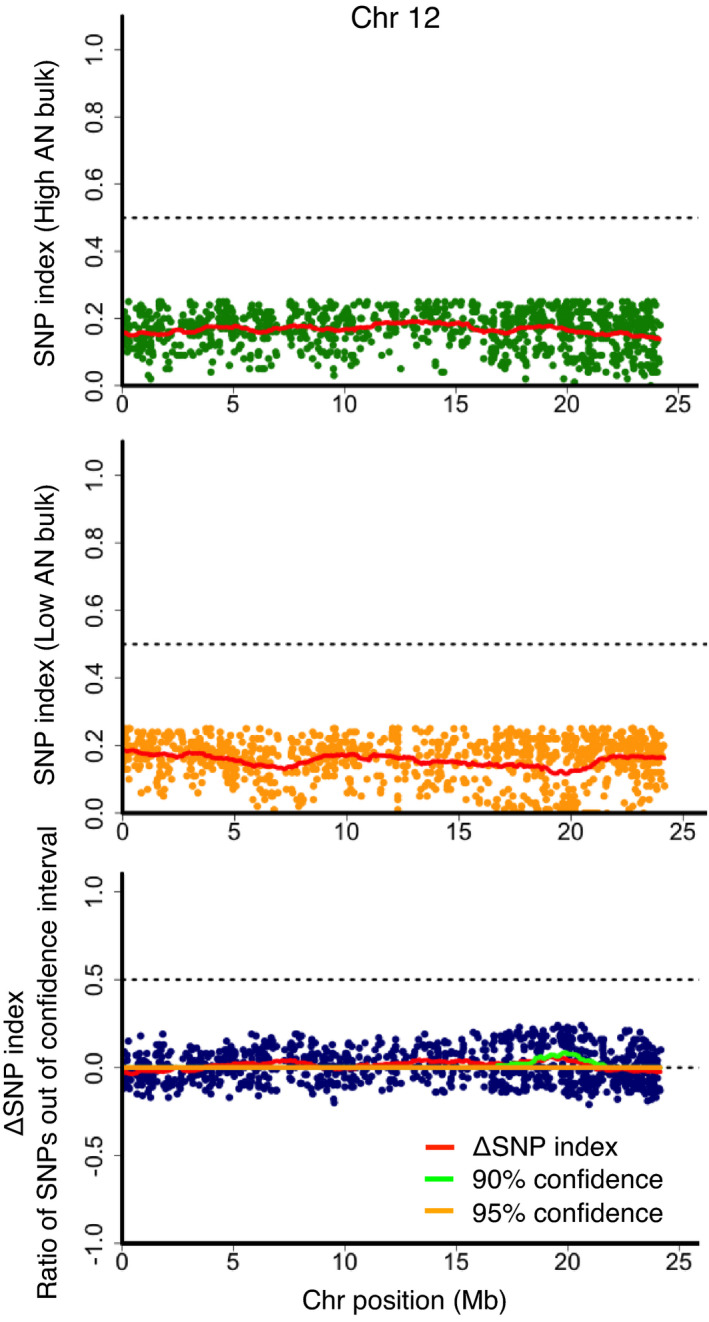
Identification of a genomic region corresponding to the sweetpotato AN locus. SNP index plots of high AN bulk (top) and low AN bulk (middle), and ΔSNP index plot with ratio of SNPs out of 90% (green line) and 95% (orange line) statistical confidence intervals under the null hypothesis of no QTLs (bottom) are depicted for Chromosome 12. Red lines indicate the sliding window average of a 2 Mb interval with a 50 kb increment for SNP index and ΔSNP index.

**Figure 5:**
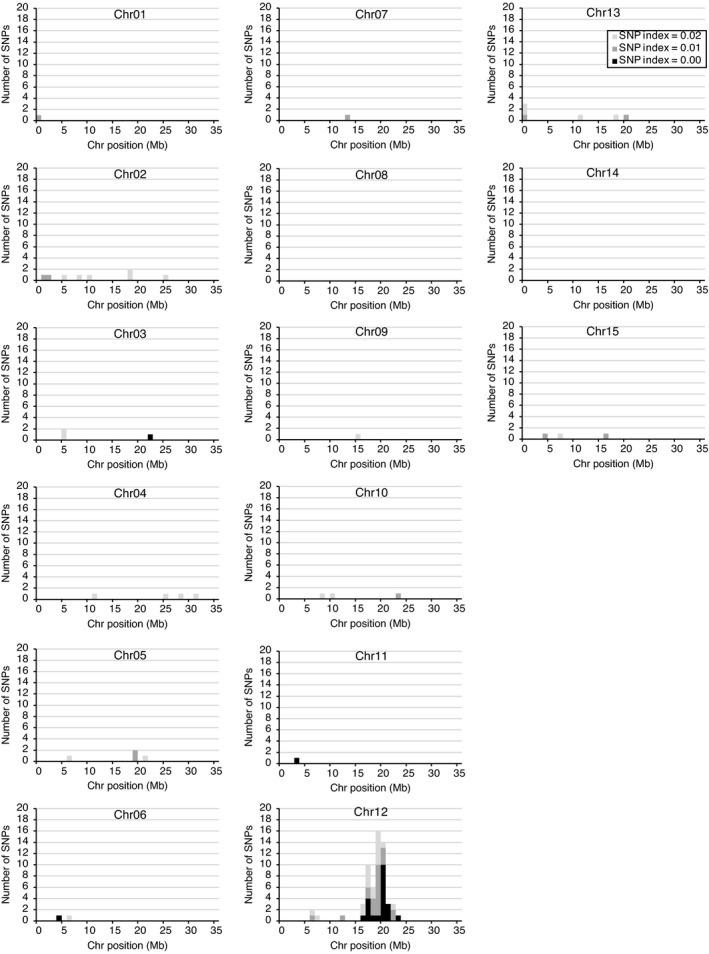
Identification of a genomic region corresponding to the sweetpotato AN locus by cluster analysis. For identification of the candidate loci, SNPs, whose SNP index for the low AN bulk was 0, 0.01 and 0.02, were extracted from the Akemurasaki‐specific simplex SNP list (Table S2, *n* = 15 430), and their distributions throughout the whole chromosomes are indicated by black, grey and light grey, respectively

The adaptability of polyploid QTL‐seq to crops with more complex genomes was verified using hexaploid and sweetpotato. In a previous study, a purple‐fleshed cultivar, Akemurasaki, was crossed with a beige‐fleshed cultivar, Konaishin, and the storage roots of their F_1_ progenies were subjected to quantification of AN content (Haque et al., 2020b). The progenies were clearly separated into 38 purple‐fleshed and 56 beige‐fleshed individuals, whose AN content in storage roots was high (A_530_ > 2.5) and low to undetectable levels (A_530_ < 0.5), respectively. In the present study, QTL‐seq was conducted with the AN content‐segregating F_1_ population. In this crossing, cv. Akemurasaki is known as a donor of the AN‐accumulating phenotype; thus, Akemurasaki reads were mapped against the reference augmented with SNPs from cv. Konaishin. Mapping with 103.1 Gb, 97.7 Gb, 100.0 Gb and 114.4 Gb reads of Akemurasaki, Konaishin, high AN bulk and low AN bulk (each consisting of 22 F_1_ individuals), corresponding to 50.3, 52.3, 52.5 and 52.5 times the coverage of the sweetpotato genome, respectively, yielded 15 430 Akemurasaki‐specific simplex SNPs. Although polyploid QTL‐seq with the simplex SNPs gave no SNPs beyond the thresholds of 95% statistical confidence, a portion of SNPs in the region of chromosome 12, 16–23 Mb presented SNP indices close to 0 for the low AN bulk read, and they skewed over 90% statistical confidence, whereas that of the high AN bulk read gave no such clustered SNPs with skewed distribution (Figure 4). Among the 24 SNPs detected with an SNP index of 0 in the low AN bulk throughout the whole genome, 21 SNPs resided in this region (Table S2). Since no other cluster showing such low AN bulk‐specific distortion of SNP index was detected throughout the genome (Figure S5), Chromosome 12, 16–23 Mb was suggested to be the sole candidate region controlling AN content. Counting the number of SNPs whose SNP indices of the low AN bulk were below 0.02 within each 1 Mb window demonstrated a single peak in the region of chromosome 12, 16–23 Mb (Figure 5). In contrast, when the analysis was conducted by exploiting the low AN parent, Konaishin‐specific simplex SNPs with the reference genome generated by Akemurasaki reads, only five SNPs were detected with an SNP index of 0 in the low AN bulk read, and they were located sparsely throughout the genome (Figure S6), confirming that the AN‐accumulating locus is inherited to F_1_ progenies by an Akemurasaki‐derived homologous chromosome. Given the larger number of SNPs with an SNP index of 0 in the low AN bulk detected with Akemurasaki‐specific simplex SNPs (24), compared to those detected with Konaishin‐specific simplex SNPs (5) (*P* < 0.001 in chi‐square test following correction for the total number of the detected SNPs, 15 430 and 15 403 for Akemurasaki‐specific and Konaishin‐specific simplex, respectively), the polyploid QTL‐seq procedure was confirmed to detect a trait‐controlling locus efficiently also in the hexaploid crop, sweetpotato.

### Development of DNA markers linked to potato *H1* and sweetpotato AN content

Since the candidate regions were detected for both *H1* locus in potato and storage root AN content‐controlling locus in sweetpotato, linked PCR markers were created at SNP sites, where SNP indices were 0 for the *h1* or low AN bulks (Table S3).

**Figure 6:**
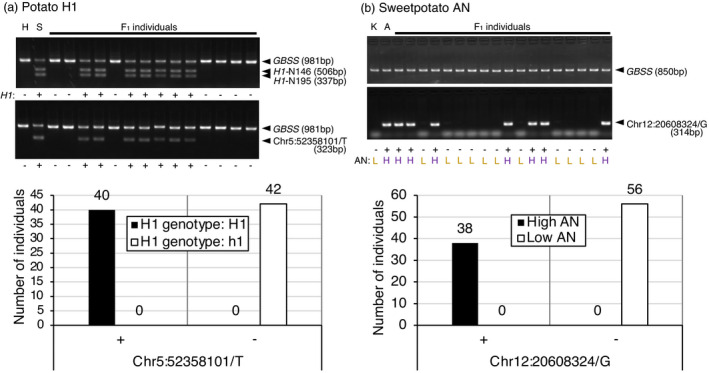
Genotyping using the developed DNA markers. (a) Evaluation of a potato *H1*‐linked SNP marker, Chr5:52358101/T. F_1_ individuals (*n* = 82) were genotyped with *H1* markers (N146 and N195) along with internal control *GBSS* primers (top). The same set of progenies were assessed for the presence (+) or absence (−) of the *H1*‐linked SNP with the Chr5:52358101/T marker (middle). The relationship between the developed marker and *H1* allele for all F_1_ individuals is shown in the graph (bottom). H: Hokkaikogane, S: Sayaka. (b) Evaluation of a sweetpotato high AN‐linked SNP marker, Chr12:20608324/G. F_1_ individuals (*n* = 94) were assessed for the presence (+) or absence (−) of the AN content‐linked SNP with the Chr12:20608324/G marker (middle) along with the control *GBSS* primers (top). Root AN content of respective individuals is indicated below as H and L for high and low content. The relationship between the developed marker and AN content for all F_1_ individuals is shown in the graph (bottom). K: Konaishin, A: Akemurasaki.

The potato *H1*‐linked marker, Chr5:52358101/T, was intended to detect the Sayaka‐specific C to T SNP, which is located on 52 358 101 bp of chromosome 5. It could be used with an internal standard *GBSS* marker by multiplexed PCR and yielded a 323 bp fragment in *H1*‐harbouring Sayaka, but not in non‐harbouring Hokkaikogane (Figure 6a). When 82 F_1_ progenies were surveyed, the marker was detected in all 40 *H1*‐possessing individuals, but the remaining individuals, which were without the resistance gene, resulted in loss of amplification. Thus, the genotypes of the Chr5:52358101/T marker completely coincided with those of the preexisting *H1* markers, N146 and N195.

The sweetpotato storage root AN content‐linked marker, Chr12:20608324/G, was used to detect the Akemurasaki‐specific C to G SNP, which is located on 20 608 324 bp of chromosome 12. Although control amplification by a *GBSS* marker in a separate PCR was necessary, it raised a 314 bp fragment in the high AN cultivar, Akemurasaki, but not in the low‐AN cultivar Konaishin (Figure 6b). When 94 F_1_ progenies were surveyed, the marker was detected in all 38 high AN individuals, and the remaining individuals (of low AN content) resulted in loss of amplification. Thus, the genotypes of the Chr12:20608324/G marker completely matched with high AN content as well as purplish root colour.

Accordingly, in both cases, completely linked DNA markers were created by exploiting SNPs in the region suggested by polyploid QTL‐seq.

## Discussion

In the present study, a new method, designated polyploid QTL‐seq, was established to identify SNPs tightly linked to agronomic traits in autopolyploid crops such as potato and sweetpotato by whole‐genome sequencing. The method is based on a bulked segregant analytical method, QTL‐seq (Takagi et al., 2013), with the following modifications. (1) Single parent‐specific simplex SNPs, which show a simple segregation ratio in the progenies, were selected and used. (2) Clusters of SNPs, which were inherited unevenly between bulked progenies showing opposite phenotypes, especially those with an SNP index of 0 for the bulk that did not display the phenotypes of interest, were explored (Figure 1). These modifications allowed for separate tracking of alleles located on each of the multiple homologous chromosomes. By applying this method, the clusters of SNPs linked to the potato cyst nematode resistance gene *H1* and storage root AN content were identified in potato and sweetpotato, respectively (Figures 3 and 5), and DNA markers linked completely to the phenotypes in the F_1_ progenies were created at the site of the presented SNPs (Figure 6). For potato *H1*, the completely linked marker was created at chromosome 5, 52 358 101 bp by alignment to the *S. tuberosum* group Phureja DM 1‐3 v6.1 reference genome. Although the exact location of *H1* has not yet been determined in the reference genome, a BLAST search with the PCR primer sequences of *H1* markers, N146 and N195, revealed that the homologous sequences were found at Chromosome 5, 52.5–52.9 Mb in the genome, according to the Spud DB website (http://solanaceae.plantbiology.msu.edu/). Therefore, it was revealed that the linked marker was created fairly in the vicinity of *H1*. For sweetpotato storage root AN content, the completely linked marker was generated at chromosome 12, 20 608 324 bp, when the alignment was performed with the publicly available *I. trifida* v3 (NSP306) reference genome. AN biosynthesis in storage roots has been reported to be regulated by the Myb transcription factor *IbMYB1* (Mano et al., 2007; Park et al., 2015; Tanaka et al., 2012). However, according to the GT4SP website (http://sweetpotato.plantbiology.msu.edu/), its closest homologues, itf12g04080.t1 and itf12g04100.t1 (with 86.2% and 87.9% nucleotide identity, respectively, suggested by a BLAST search of the *IbMYB1* genomic sequence; accession number; AB258985), were located on chromosome 12, 2.4–2.5 Mb in the *I. trifida* v3 (NSP306) reference genome, which is distant from the QTL detected in the present study. Several other genes encoding Myb domain proteins reside in the QTL candidate region, chromosome 12, 16–24 Mb. An Myb‐related gene or an unidentified gene might be the causative gene for AN content. Alternatively, *MYB1* might not exist in the genome of *I. trifida*, which does not accumulate AN in the root; thus, it would not be included within the candidate region of the reference genome.

Even though the cost of NGS has been tremendously reduced, it still occupies a significant proportion of expenditure for polyploid QTL‐seq. Therefore, the minimum number of reads necessary for the analysis was verified. In the case of potato *H1*, 52.5 Gb reads of the respective samples, parents and two separate bulks were sufficient to determine the candidate region (Figure S4). If a phenotype of interest is estimated to be regulated by a single gene from its segregation ratio of 1:1 for progenies of presence versus absence of the phenotype, as in the case of potato *H1*, analysis with read data of ˜50 Gb might be feasible, since many SNPs with an SNP index of nearly 0 would be detected in a clear cluster. However, in case that involvement of more than one gene was expected from a continuous segregation pattern in the progenies’ phenotypic value, read data > 100 Gb would be recommended to distinguish skewed SNPs whose SNP index would not reach 0. In the case of potato, 100 Gb reads would render an aligned read depth of approximately 48, which affords sufficiently precise evaluation of SNP index, whereas 50 Gb reads are not sufficient to distinguish slight differences, such as that of the second decimal place in the SNP index. In the current NGS apparatus, such as HiSeq X and NovaSeq6000, the cost for a 100 Gb read, which is equivalent to data obtained from one lane of HiSeq X, is not much different from that for a 50 Gb read. The >100 Gb read would be the most suitable choice to accomplish polyploid QTL‐seq.

Recently, several methods for mapping QTLs using NGS have been developed for autopolyploid crops. RenSeq and GenSeq are based on bulked segregant analyses, which use sequence‐captured libraries targeting arbitrarily selected, nucleotide binding‐site leucine‐rich repeat resistance genes, and single/low‐copy number genes dispersed throughout the potato genome, respectively. In an analysis of tetraploid potato, RenSeq utilized 3314 SNPs that conformed to the expected allele frequency of the simplex (Strachan et al., 2019), and GenSeq exploited 1980 single/low‐copy number genes, which had been selected for sequence capture beforehand (Chen et al., 2018). Both methods yielded SNPs that were inherited unevenly between progenies with and without potato cyst nematode resistance as a cluster (Strachan et al., 2019). On the other hand, RAD‐seq based methods were developed for QTL and genome‐wide association analyses in hexaploid sweetpotato. QTLs for root‐knot nematode resistance and storage root β‐carotene content were detected through analyses with 5,563 and 5,952 donor parent‐derived SNPs for their expected segregation ratios for simplex, respectively (Haque et al., 2020a; Sasai et al., 2019), and a QTL for AN content in storage roots was presented by an analysis of 15 747 simplex SNPs (Haque et al., 2020b). QTLs for root‐knot nematode resistance and storage root β‐carotene content were also detected by another reduced representation sequencing‐based platform that consisted of GBSpoly, MAPpoly and QTLpoly (Gemenet et al., 2020; Oloka et al., 2021). Another approach is the sequencing of individual progenies with low quantity of read data. Yamamoto et al. (2020) developed a low‐coverage RAD‐seq‐based genotyping method, ngsAssocPoly, and detected loci associated with skin colour and internode length by calculating allele dosage probabilities. Chen et al. (2021) created OutcrossingSeq, in which 1807 F_1_ progenies were barcoded and whole‐genome resequenced with 1.5‐time coverage for each individual to identify loci controlling vein colour, leaf shape and number of storage roots. These low‐coverage NGS methods are cost‐efficient but labour‐intensive because the preparation of barcoded sequencing libraries is necessary for each progeny. Recently, a mapping method exploiting *k*‐mers, namely comparative subsequence set analysis (CoSSA), was developed (Prodhomme et al., 2019). This method is based on bulked segregant analysis, in which bulk‐specific *k*‐mers are extracted by subtracting *k*‐mers of one bulk from those of the other bulk and are aligned to the reference genome to determine genomic regions associated with the traits of interest. In this method, the genomic region that is unique to one bulk is directly indicated by the alignment of the bulk‐specific *k*‐mer. Using this method, a potato wart disease resistance gene and a self‐compatibility‐associated gene have been identified in potato (Clot et al., 2020; Prodhomme et al., 2019). This *k*‐mer‐based method is effective in the exploration of genomic fragments that have been incorporated from donor parents, such as wild relatives. In contrast, SNP‐based methods, including polyploid QTL‐seq, are versatile not only to capture such incorporated genes by detecting linked SNPs in the vicinity of the incorporated site, but also to grasp genes that exist in the genomes of both parents but harbour varying sequences.

Compared to the above methods, polyploid QTL‐seq has the following advantages. (1) Although a reference genome is necessary, complicated procedures for the preparation of sequencing libraries for hundreds of progenies and the construction of elaborate genetic linkage maps are not required. Therefore, polyploid QTL‐seq can be easily applied to new progenies developed by crossing unexplored materials. (2) Alignment with whole‐genome sequence reads yields profiles of up to 100 240 and 15 430 simplex SNPs throughout the genome without any biased selection for the present potato and sweetpotato materials, which enables the creation of tightly linked DNA markers in the vicinity of the causative allele. (3) Since many linked SNPs are present in a given candidate region, there would be a choice of several SNPs for the arrangement of DNA markers. Thus, even if cultivars other than those used to establish DNA markers are subjected to breeding and do not show polymorphism for the SNP used as the DNA marker, it would be possible to exploit the other neighbouring SNPs for preparation of available DNA markers, as long as the same donor parent is used.

In conclusion, polyploid QTL‐seq is a versatile method that is free from specialized manipulation for sequencing and the construction of elaborate linkage maps and enables rapid development of tightly linked DNA markers in the autopolyploid crops, potato and sweetpotato. This method might be applicable to other autopolyploid crops such as blueberries, sugarcane and roses. In the present study, tightly linked DNA markers were created in cases of traits regulated by a single locus. However, the ability to detect QTLs in cases of multiple gene‐regulated traits remains to be verified. Further validation with agronomically important traits, such as disease resistance and tuber quality, which are thought to be under the control of multiple genes, is anticipated.

## Experimental procedures

### Plant materials

Potato *H1*‐segragating populations were generated by crossing *S*. *tuberosum* cv. Hokkaikogane (*H1* nulliplex; *h1*/*h1*/*h1*/*h1*), and cv. Sayaka (*H1* simplex; *H1*/*h1*/*h1*/*h1*), herein designated as P1 and P2, respectively. F_1_ seeds were sown on horticultural soil (Takii, Kyoto, Japan) and grown in an air‐conditioned greenhouse. The leaves of 82 progenies and their parents were used for the extraction of genomic DNA.

A sweetpotato storage root AN content‐segregating population was used in a previous study (Haque et al., 2020b). Briefly, F_1_ progenies generated by crosses of *I*. *batatas* cv. Konaishin (low AN content; 0.10 of absorbance at 530 nm) and cv. Akemurasaki (high AN content; 12.01 of absorbance at 530 nm), herein designated P1 and P2, respectively, were grown in plastic pots in a greenhouse. The storage roots of 94 progenies and their parents were harvested, and their AN content was determined as reported previously (Haque et al., 2020b). Unexpanded leaves at the shoot apex of the same individuals were used for extraction of genomic DNA.

### Genomic DNA extraction, bulking and sequencing

Potato genomic DNA was extracted using a DNeasy Plant Mini Kit (QIAGEN, Hilden, Germany). To detect the *H1* allele, PCR mixtures (total 10 μL) containing 1× GoTaq Colorless Master Mix (Promega, Madison, WI), 10 ng of genomic DNA, and 3 pmol each of forward and reverse primers of *H1*‐N146, *H1*‐N195 and *GBSS* (as an internal control) (Table S3) were used for PCR with the following programme: 95 °C for 2 min, followed by 30 cycles of 95 °C for 30 s, 55 °C for 30 s, 72 °C for 1 min, and a final extension period of 72 °C for 2 min. The amplicons were verified by electrophoresis on a 1.5% agarose gel. By combining an equal amount of DNA from each of the 23 F_1_ individuals of *H1* and *h1* (*H1* absent) genotypes, bulked DNA samples were prepared.

Sweetpotato genomic DNA was extracted using ISOSPIN Plant DNA (Nippon Gene, Tokyo, Japan). By combining an equal amount of DNA from each of 22 F_1_ individuals with high (7.44 to 15.43 absorbance at 530 nm) and low (0.11 to 0.25 absorbance at 530 nm) AN content according to AN quantity determined in a previous study (Haque et al., 2020b), the respective bulked DNA samples were prepared.

The sequencing library was constructed using a TruSeq DNA PCR‐Free Sample Preparation Kit (Illumina, San Diego, CA) and subjected to 150 bp paired end sequencing using an Illumina NovaSeq6000 DNA sequencer. The FASTQ sequence data are available from the DDBJ Sequence Read Archive under the following accession numbers: DRA011095 and DRA011096 for potato, and DRA011381 for sweetpotato.

### Polyploid QTL‐seq analysis

Polyploid QTL‐seq analysis was performed with the QTL‐seq v1.4.4 pipeline, which was downloaded from Iwate Biotechnology Research Center (http://genome‐e.ibrc.or.jp/home/bioinformatics‐team/mutmap), using an iMac Pro computer equipped with 36‐thread Intel Xeon W processor, 128 GB RAM, 4 TB HDD. The following programmes were installed to run the pipeline: Perl v5.28.2, R version 4.0.2, BWA version 0.7.17‐r1188, SAMtools version 0.1.8 and FASTX Toolkit version 0.0.14. The schematics are shown in Figure 1. The analysis was conducted with two separate QTL‐seq pipelines: one for comparison of SNP indices of two bulks showing opposite phenotypes and the other for extraction of P2 parent‐specific simplex SNPs, with the following modification of the configuration parameters from the default setting: Key3_Maximum_mismatches of 4, Key3_Minimum_number_of_depth_for_bulked of 10, Key3_Maximum_number_of_depth_for_bulked of 500 (in potato) or 800 (in sweetpotato), Key5_Miss of 4, Key5_Cutoff of 0.0, Key5_Population of F2, Key5_Depths of 7, Key5_Min_snpindex of 0.0, and Key5_Max_snpindex of 0.36 (in potato) or 0.25 (in sweetpotato). Both pipelines were started by preparing the same P1 parent reference sequence by replacing the publicly available reference genome sequence, *S. tuberosum* group Phureja DM 1‐3 v6.1 (Pham et al., 2020) or *I. trifida* v3 (NSP306) (Wu et al., 2018), with SNPs detected on P1 read alignment. Then, the P1 short reads were self‐aligned to the yielded reference sequence again to exclude the detected SNPs from the analysis as spurious SNPs (Filter 1). In the first pipeline, the reads of two bulks showing opposite phenotypes were aligned to the P1 reference sequence to generate a merged SNP list including read counts and SNP index of both bulks for respective SNPs (generated in the 4.search_for_pair/40.merge_paired directory in the pipeline). Then, the SNPs that matched with the following criteria: read counts of ≥40 and SNP index of ≤0.36 for both bulks in case of potato or read counts of ≥50 and SNP index of ≤0.25 for both bulks in case of sweetpotato (unless otherwise stated) were extracted from the list to select simplex SNPs (Filter 2). In the second pipeline, P1 and P2 reads were aligned to the P1 reference sequence to obtain pileup lists of SNPs detected for respective reads (snp.pileup files generated in the 3.alignment/30.coval_call directory in the pipeline). After combining common SNPs in the pileup lists for both parents in the second pipeline with those in the above merged SNP list of the bulk reads in the first pipeline using the VLOOKUP function in Microsoft Excel, P2‐specific simplex SNPs were selected with the following criteria: read counts of ≥40 and SNP index of 0.10–0.36 on P2 alignment in the case of potato, or read counts of ≥50 and SNP index of 0.08–0.25 on P2 alignment in the case of sweetpotato, unless otherwise stated, as well as an SNP index of 0 on P1 alignment (Filter 3). Finally, the list of SNPs that passed through the above filters was placed in the 4.search_for_pair/40.merge_paired directory in the first pipeline and used for the subsequent sliding window analysis to yield the average SNP index and *P* value using Fisher’s exact test for respective SNPs with 2 Mb window size and a 50 kb increment.

For mining cluster(s) of SNPs linked to the phenotype of interest, SNPs whose SNP indices of the *h1* or low AN bulks were 0, 0.01 and 0.02 were counted in the above filtered P2‐specific simplex SNP list, and their numbers in given 1 Mb intervals are indicated as histograms for respective chromosomes.

To evaluate the efficacy of the developed method, the same analysis was conducted with P1 and P2 parents exchanged, and a chi‐square test was performed for the number of SNPs presented as candidates.

### Preparation and evaluation of linked DNA makers

To prepare tightly linked DNA markers, DNA primers were designed at SNP sites in the middle of the candidate region identified by QTL‐seq analysis (Table S3). To evaluate the potato *H1*‐linked DNA marker, PCR mixtures (total 10 μL) containing 1× GoTaq Colorless Master Mix, 10 ng of genomic DNA of each F_1_ individual, and 3 pmol each of forward and reverse primers of Chr5:52358101/T and *GBSS* (as an internal control) (Table S3) were used for PCR with the following programme: 95 °C for 2 min, followed by 30 cycles of 95 °C for 30 s, 55 °C for 30 s, 72 °C for 1 min, and a final extension period of 72 °C for 2 min. The amplicons were verified by electrophoresis on a 1.5% agarose gel. For evaluation of the DNA marker linked to the sweetpotato AN content‐regulating QTL, PCR mixtures (total 10 μL) containing 1× GoTaq Colorless Master Mix, 10 ng of genomic DNA of each F_1_ individual, and 5 pmol each of forward and reverse primers of Chr12:20608324/G or *GBSS* (as a control; Table S3) were used for PCR with the following programme: 95 °C for 2 min, followed by 30 cycles of 95 °C for 30 s, 55 °C for 30 s, 72 °C for 1 min, and a final extension period of 72 °C for 2 min. The amplicons were verified by electrophoresis on a 1.5% agarose gel.

## Accession numbers

The FASTQ sequence data are available from the DDBJ Sequence Read Archive under accession numbers DRA011095, DRA011096 and DRA011381.

## Conflicts of interest

The authors declare no conflict of interests.

## Author contributions

HY and HT conceived the research project and designed the experiments. EH and MT created the sweetpotato F_1_ population and quantified the AN content in their storage roots. KA, ES, KA, SO, KK and ST created the potato F_1_ population. HY conducted polyploid QTL‐seq and linked DNA marker analyses and wrote the manuscript.

## Supporting information

**Figure S1** Simulation test for obtaining a 95% confidence interval assuming simplex SNPs.
**Figure S2** Plots of SNP index, ΔSNP index and ratio of SNPs out of confidence intervals generated by polyploid QTL‐seq analysis with an *H1*‐segregating potato F_1_ population.
**Figure S3** Cluster plot of polyploid QTL‐seq analysis using the Sayaka reference sequence and Hokkaikogane‐specific simplex SNPs.
**Figure S4** Identification of a genomic region corresponding to the potato H1 locus by cluster analysis using polyploid QTL‐seq data with a reduced number of reads.
**Figure S5** Plots of SNP index, ΔSNP index, and ratio of SNPs out of confidence intervals generated by polyploid QTL‐seq analysis with the AN‐segregating sweetpotato F1 population.
**Figure S6** Cluster plot of QTL‐seq analysis using the Akemurasaki reference sequence and Konaishin‐specific simplex SNPs.

**Table S1** Sayaka‐specific simplex SNPs identified by the polyploid QTL‐seq analysis of potato *H1*‐segregating progeny.
**Table S2** Akemurasaki‐specific simplex SNPs identified by the polyploid QTL‐seq analysis of sweetpotato AN‐segregating progeny.
**Table S3** Primers used in the present study.
