# Supplementary material for: Polyploid QTL‐seq towards rapid development of tightly linked DNA markers for potato and sweetpotato breeding through whole‐genome resequencing
Source: Plant Biotechnol J. 2021 Jun 2;19(10):2040–51. doi: 10.1111/pbi.13633 (PMC8486255; doi:10.1111/pbi.13633)
Supplement: Supplementary file 1 — Figure S1 Simulation test for obtaining a 95% confidence interval assuming simplex SNPs. Figure S2 Plots of SNP index, ΔSNP index and ratio of SNPs out of confidence intervals generated by polyploid QTL‐seq analysis with an H1‐segregating potato F1 population. Figure S3 Cluster plot of polyploid QTL‐seq analysis using the Sayaka reference sequence and Hokkaikogane‐specific simplex SNPs. Figure S4 Identification of a genomic region corresponding to the potato H1 locus by cluster analysis using polyploid QTL‐seq data with a reduced number of reads. Figure S5 Plots of SNP index, ΔSNP index, and ratio of SNPs out of confidence intervals generated by polyploid QTL‐seq analysis with the AN‐segregating sweetpotato F1 population. Figure S6 Cluster plot of QTL‐seq analysis using the Akemurasaki reference sequence and Konaishin‐specific simplex SNPs. [file PBI-19-2040-s002.pdf]

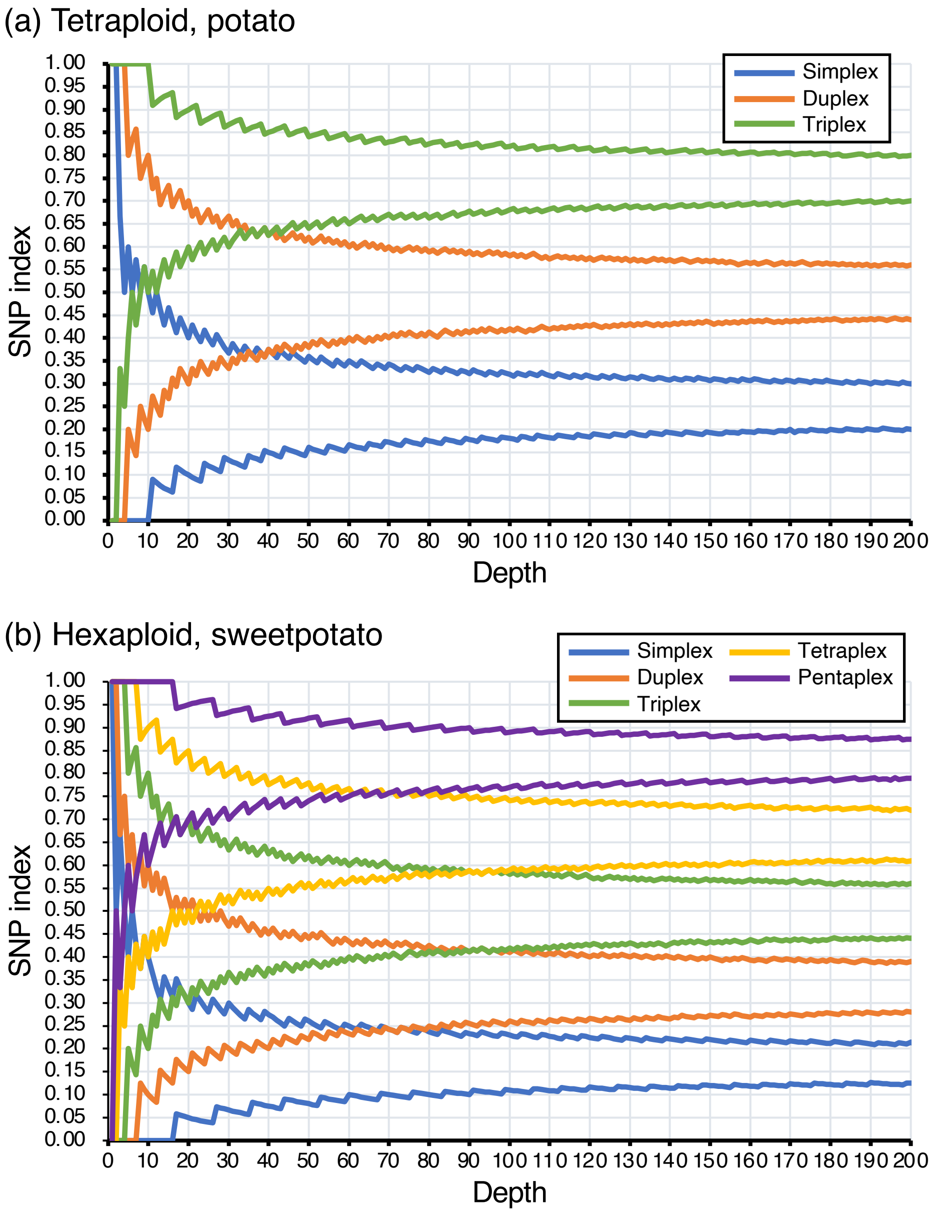

Figure S1 Simulation test for obtaining a 95% confidence interval assuming simplex SNPs. The 95% confidence interval of SNP index value is calculated by simulation of 10,000 replications assuming simplex (blue), duplex (orange), triplex (green), tetraplex (yellow), pentaplex (purple) SNPs at a given depth in case of tetraploid, potato (a) and hexaploid, sweetpotato (b). Lower and upper limits are indicated with lines in respective colors.

(a)

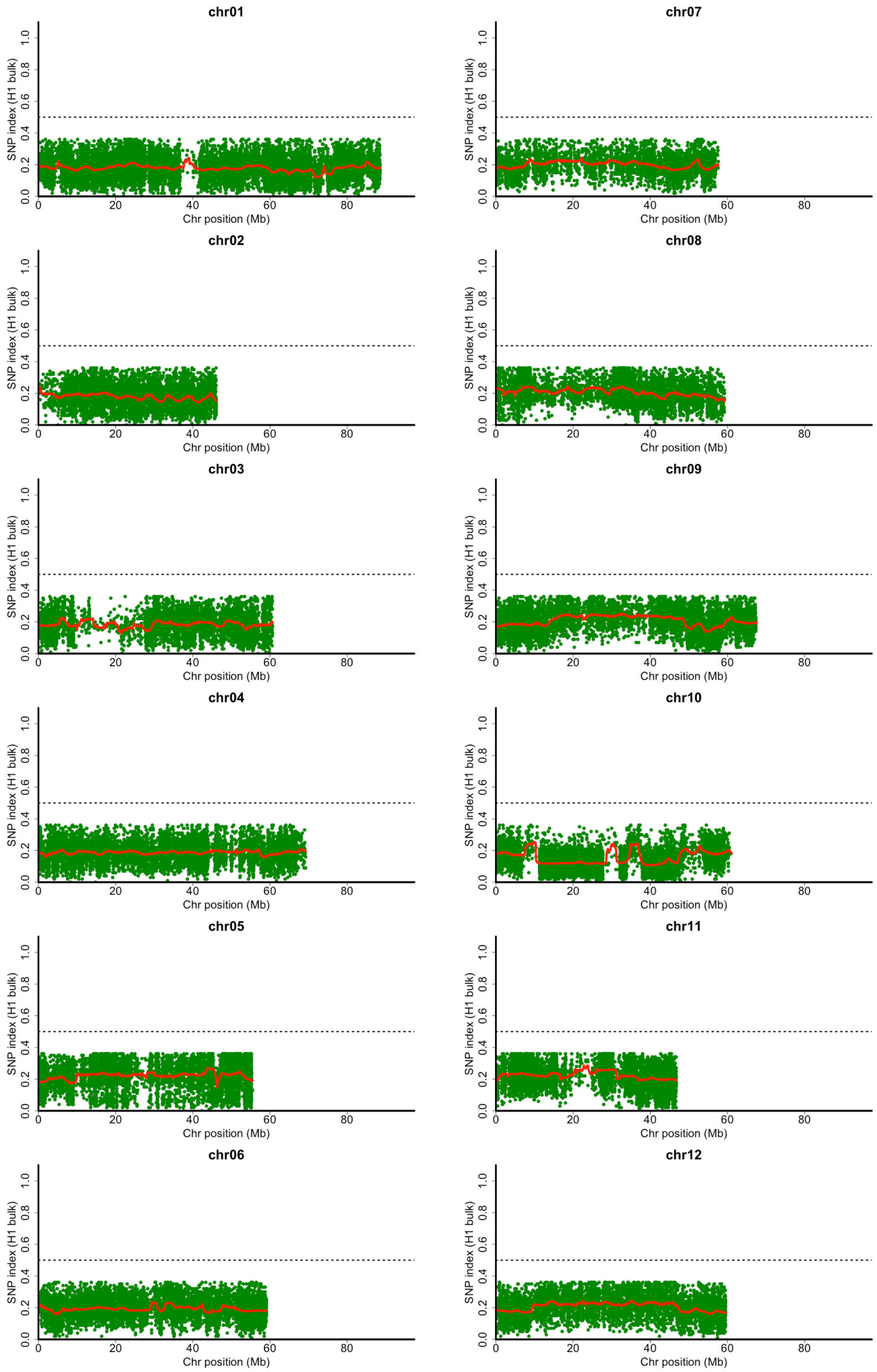

Figure S2 Plots of SNP index, ΔSNP index, and ratio of SNPs out of confidence intervals generated by polyploid QTL-seq analysis with the *H1*-segregating potato F_1 population. (a) SNP index of the *H1* bulk. (b) SNP index of the *h1* bulk. (c) ΔSNP index calculated by subtraction of the index of *h1* bulk from that of *H1* bulk, along with the ratio of SNPs out of 95% (green line) and 99% (orange line) statistical confidence intervals under the null hypothesis of no QTLs. Red lines indicate average value obtained by the sliding window analysis of 2 Mb intervals with 50 kb increments.

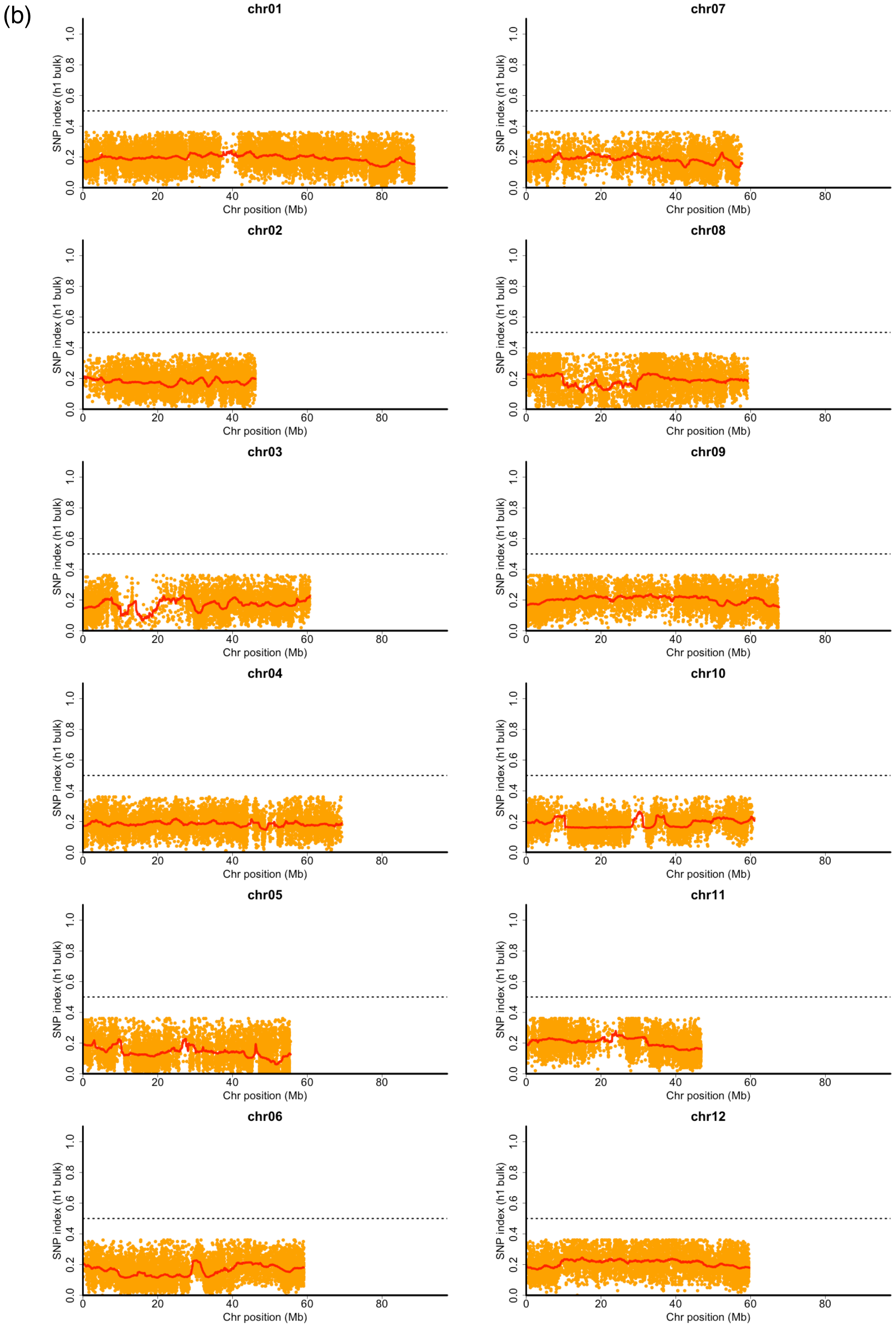

Figure S2 (continued)

(c)

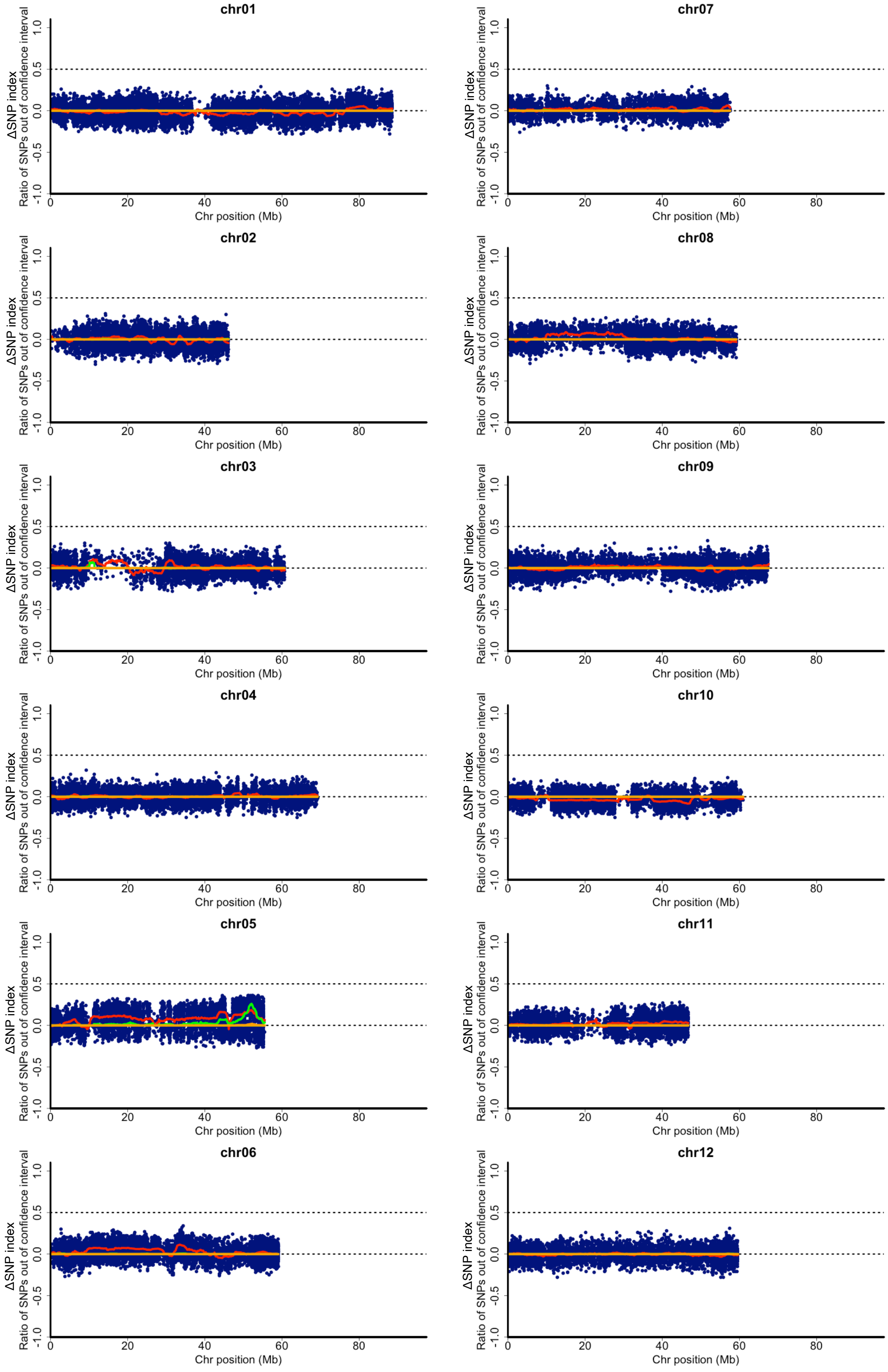

Figure S2 (continued)

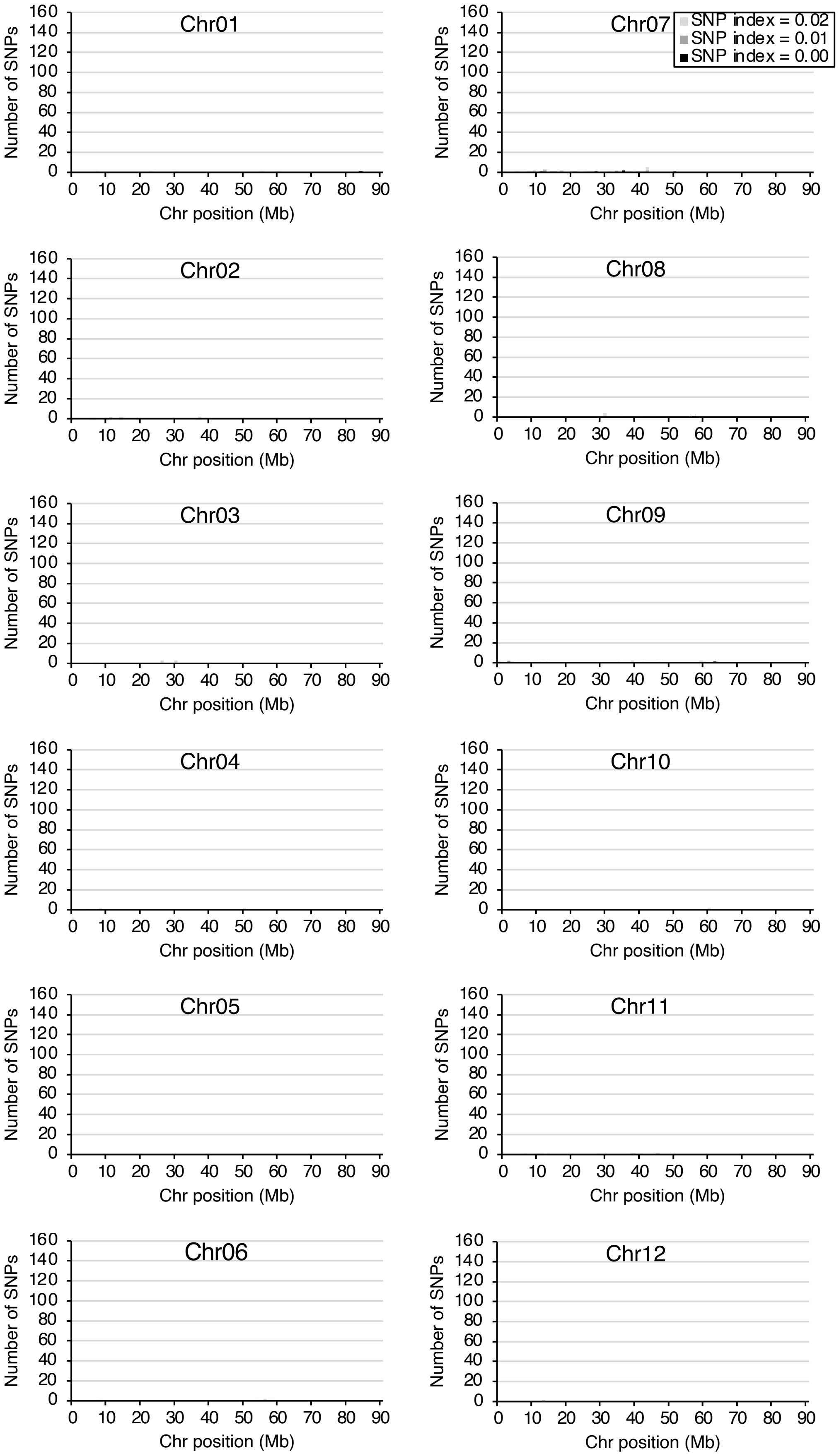

Figure S3 Cluster plot of polyploid QTL-seq analysis using the Sayaka reference sequence and Hokkaikogane-specific simplex SNPs. SNPs (with a SNP index for the *h1* bulk of 0, 0.01, and 0.02) were extracted from Hokkaikogane-specific simplex SNPs (n = 71,083), and their distributions throughout the whole chromosomes are indicated by black, gray, and light gray, respectively.

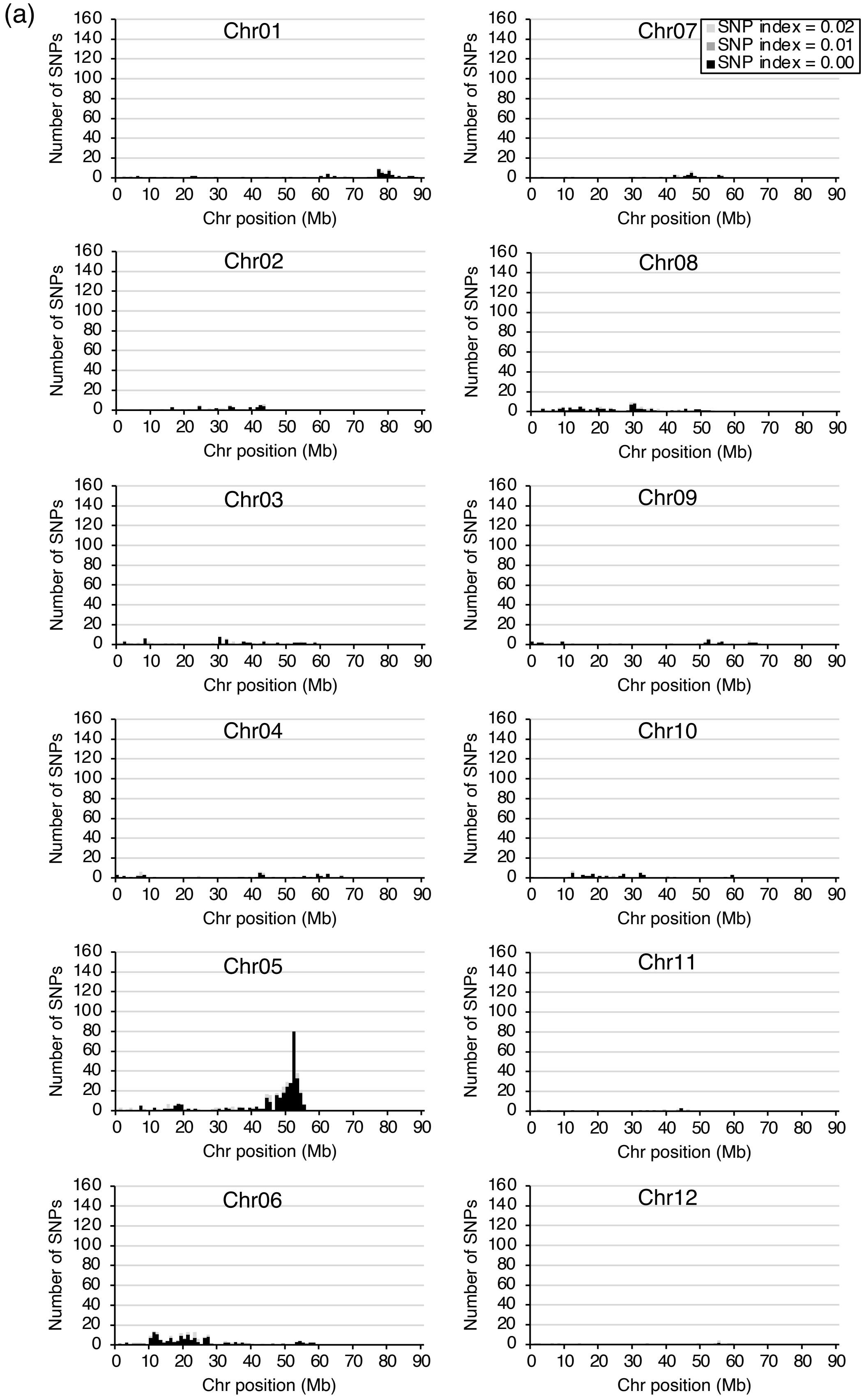

Figure S4 Identification of a genomic region corresponding to the potato *H1* locus by cluster analysis using polyploid QTL-seq data with a reduced number of reads. (a) The cluster analysis using a half amount (52.5 Gb) of the obtained raw reads with SNP extraction depth filter of ≥ 25. (b) The cluster analysis using one quarter amount (26.25 Gb) of the obtained raw reads with SNP extraction depth filter of ≥ 15. SNPs (with a SNP index for the *h1* bulk of 0, 0.01, and 0.02) were extracted from the Sayaka-specific simplex SNPs [(a) n = 78,598 and (b) n = 33,609], and their distributions throughout the whole chromosomes are indicated by black, gray, and light gray, respectively.

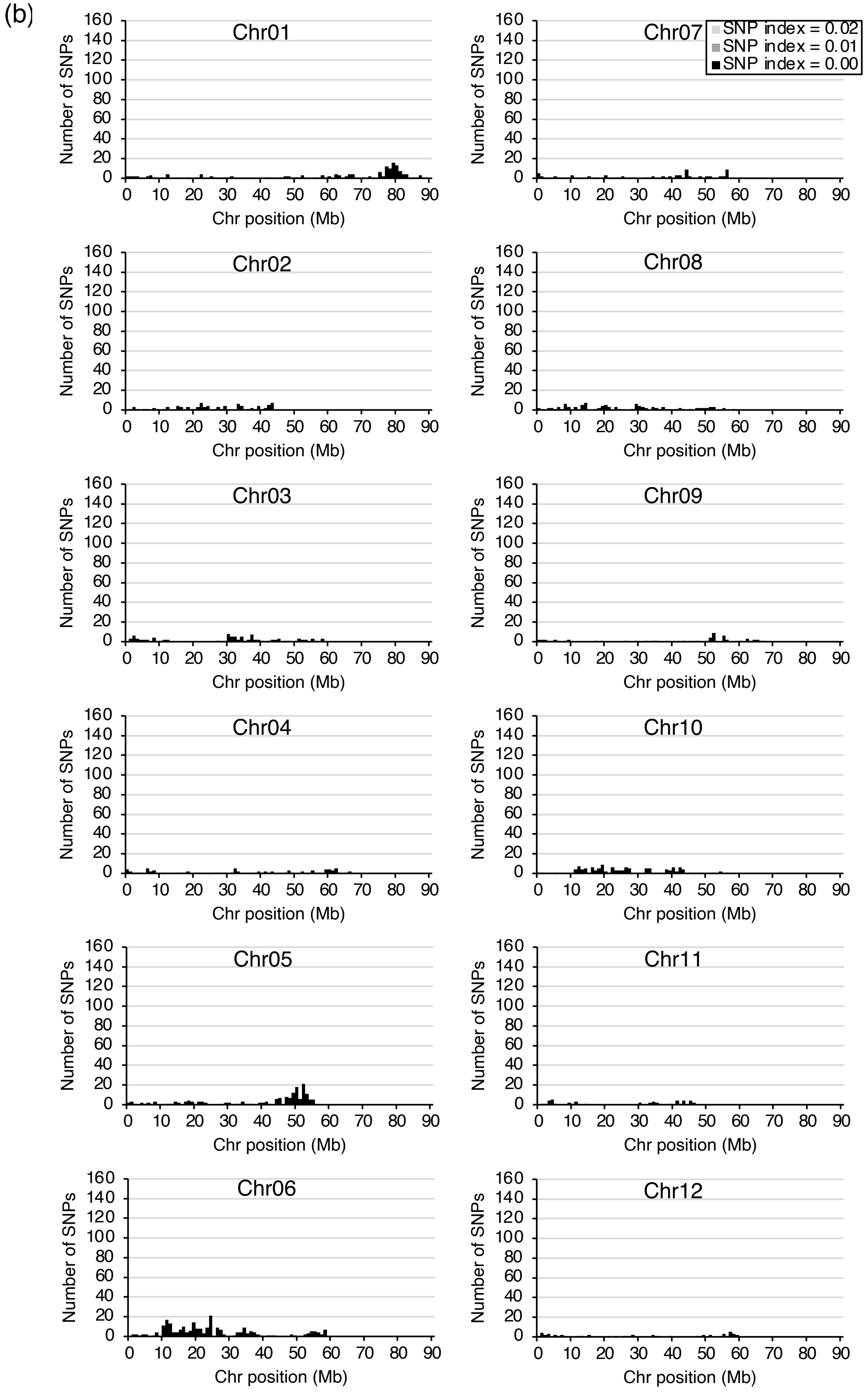

Figure S4 (continued)

(a)

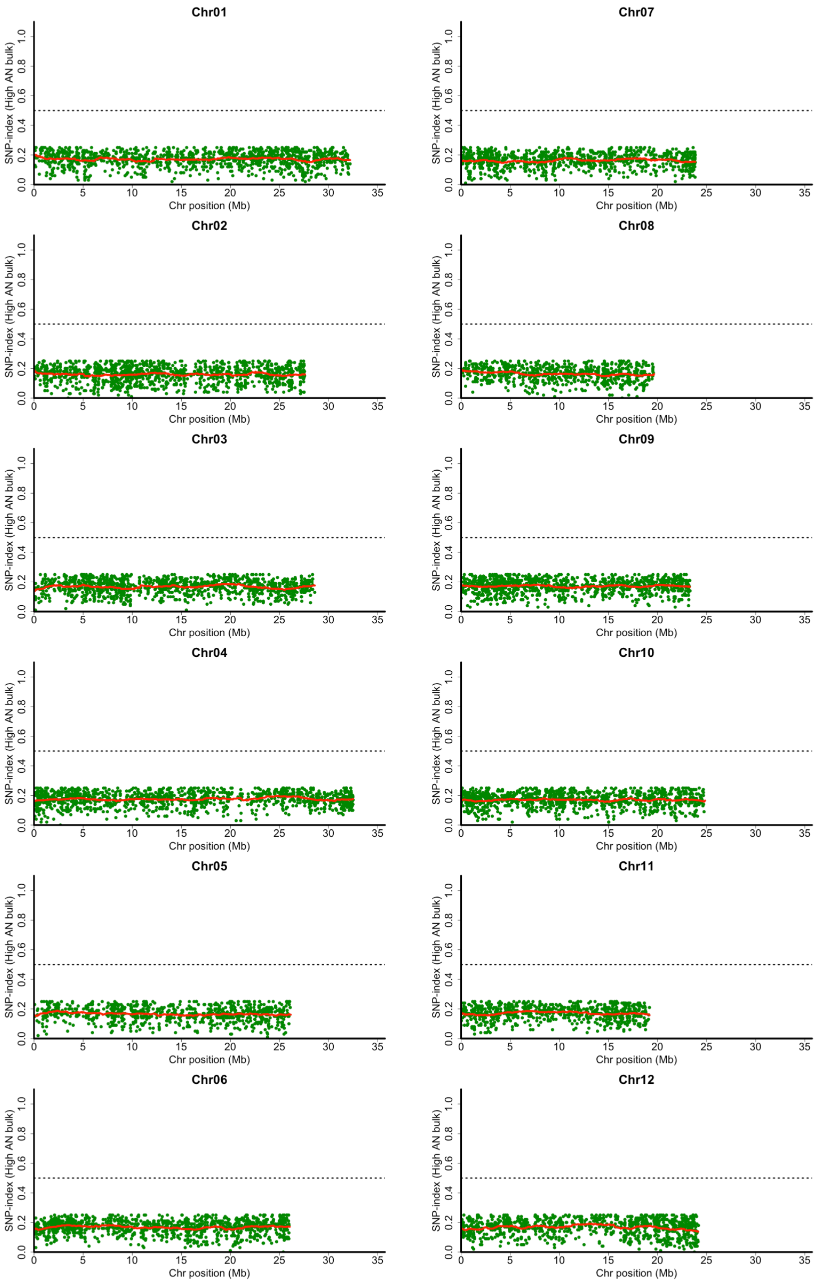

Figure S5 Plots of SNP index, ΔSNP index, and ratio of SNPs out of confidence intervals generated by polyploid QTL-seq analysis with the AN-segregating sweetpotato F_1 population. (a) SNP index of the high AN bulk. (b) SNP index of the low AN bulk. (c) ΔSNP index calculated by subtraction of the index of low AN bulk from that of high AN bulk, along with the ratio of SNPs out of 90% (green line) and 95% (orange line) statistical confidence intervals under the null hypothesis of no QTLs. Red lines indicate the average value obtained by the sliding window analysis of 2 Mb intervals with 50 kb increments.

(b)

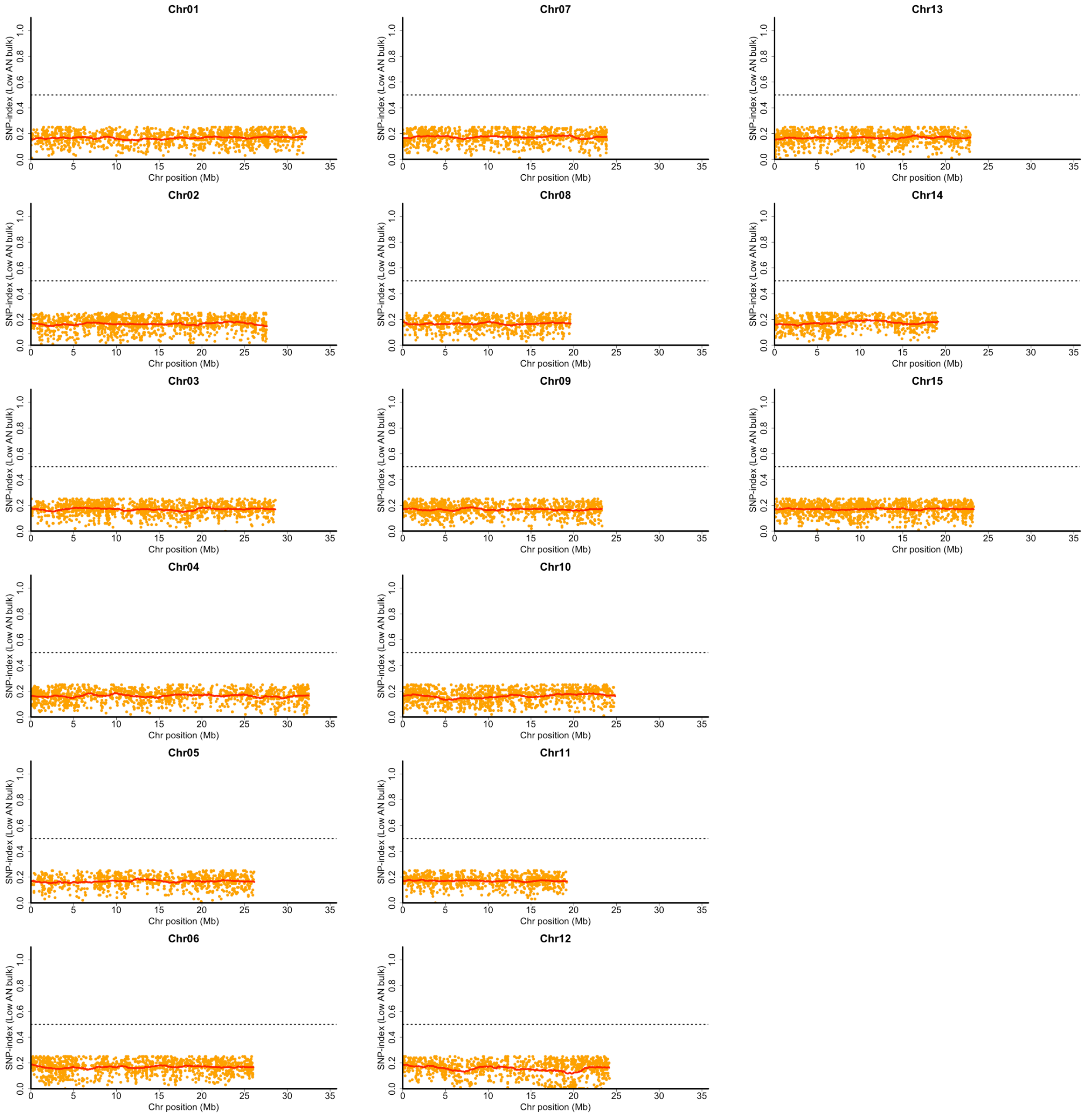

Figure S5 (continued)

(c)

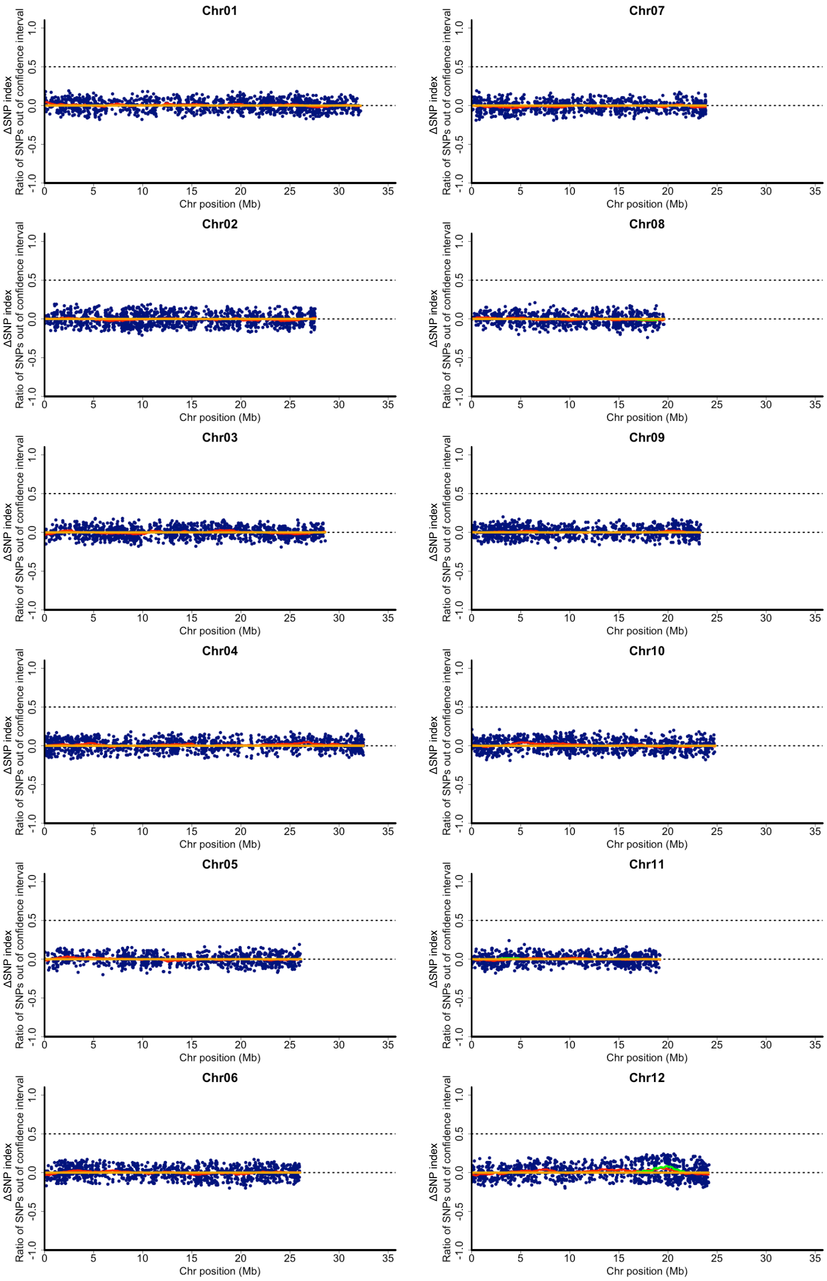

Figure S5 (continued)

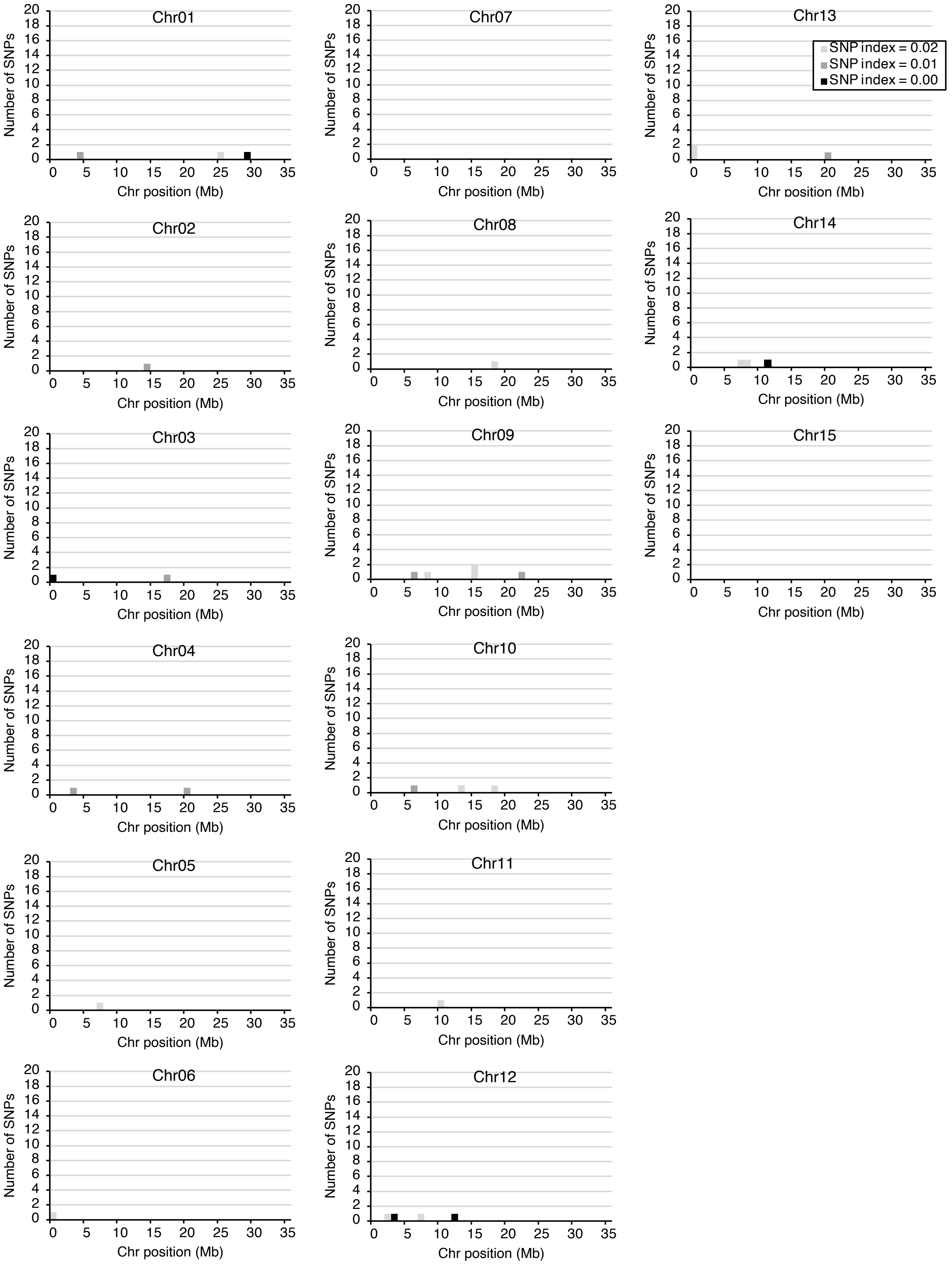

Figure S6 Cluster plot of QTL-seq analysis using the Akemurasaki reference sequence and Konaishin-specific simplex SNPs. SNPs (with SNP index for the low AN bulk of 0, 0.01, and 0.02) were extracted from Konaishin-specific simplex SNPs (n = 15,403), and their distributions throughout the whole chromosomes are indicated by black, gray, and light gray, respectively.
